# Children with Hashimoto’s Thyroiditis Have Increased Intestinal Permeability: Results of a Pilot Study

**DOI:** 10.4274/jcrpe.galenos.2020.2019.0186

**Published:** 2020-09-02

**Authors:** Banu Küçükemre Aydın, Melek Yıldız, Abdurrahman Akgün, Neval Topal, Erdal Adal, Hasan Önal

**Affiliations:** 1 University of Health Sciences Turkey, Kanuni Sultan Süleyman Training and Research Hospital, Unit of Pediatric Endocrinology and Metabolism, İstanbul, Turkey; 2 Medipol University Faculty of Medicine, Department of Pediatric Endocrinology and Metabolism, İstanbul, Turkey

**Keywords:** Autoimmune thyroiditis, congenital hypothyroidism, zonulin, increased intestinal permeability

## Abstract

Increased intestinal permeability (IIP) precedes several autoimmune disorders. Although Hashimoto’s thyroiditis (HT) is the most common autoimmune disorder, the role of IIP in its pathogenesis had received little attention. Zonulin plays a critical role in IIP by modulating intracellular tight junctions. Rise of serum zonulin levels were shown to indicate IIP in human subjects. In this case-control study, we examined the hypothesis that patients with HT have IIP. We studied 30 children and adolescents with HT, and 30 patients with congenital hypothyroidism (CH) matched for age, gender and body mass index (BMI). Serum zonulin levels, free thyroxine (fT4), thyroid stimulating hormone (TSH), anti-thyroglobulin antibody and anti-thyroid peroxidase antibody were measured. Zonulin levels were significantly higher in patients with HT than patients with CH (59.1±22.9 ng/mL vs. 43.3±32.9 ng/mL, p=0.035). In patients with HT, zonulin levels were positively correlated with weight (r=0.406, p=0.03), BMI (r=0.486, p=0.006) and levothyroxine dose (r=0.463, p=0.02). In patients with CH, zonulin levels were positively correlated with age (r=0.475, p=0.008), weight (r=0.707, p<0.001), BMI (r=0.872, p<0.001) and levothyroxine dose (r=0.485, p=0.007). After adjusting for age, weight, TSH and fT4 levels, serum zonulin was only associated with levothyroxine dose in patients with HT (R2=0.36, p=0.05). In patients with CH, only weight was associated with zonulin levels (R2=0.62, p<0.001). In conclusion, higher zonulin levels in children and adolescents with HT suggested IIP in these patients. Additionally, the association between zonulin levels and levothyroxine dose might imply a relationship between serum zonulin and disease severity.

**What is already known on this topic?**

Increased intestinal permeability (IIP) was shown to precede the onset of several autoimmune disorders. Although Hashimoto’s thyroiditis (HT) is the most common autoimmune disorder, the role of IIP in its pathogenesis had received little attention. Exact disease mechanisms are poorly elucidated in HT.

**What this study adds?**

Children and adolescents with HT had increased zonulin levels compared to their age, gender and body mass index matched peers with congenital hypothyroidism. Higher zonulin levels suggested IIP in these patients.

## Introduction

Both genetic predisposition and environmental factors play some role in triggering Hashimoto thyroiditis (HT), but the exact mechanisms are still not fully understood. Increased intestinal permeability (IIP) was shown to be a constant and early feature of several autoimmune disorders (1). Although HT is the most common autoimmune disorder worldwide, the role of IIP in its pathogenesis had received little attention.

Human zonulin regulates intestinal permeability by disassembling the tight junctions (TJ) (2). Zonulin was shown to play a key role in IIP, when up regulated (3). Increased zonulin levels were reported to be correlated with IIP *in vivo* and changes in claudin-1, claudin-2, and myosin *IXB* gene expression (4). Higher zonulin expression was reported in the intestinal tissues of patients with many autoimmune disorders (4). Increased serum zonulin levels were detected in human subjects during the pre-diabetic stage and preceded the onset of type 1 diabetes (4). In a rat model, zonulin-dependent IIP was shown to precede the onset of type 1 diabetes by 2-3 weeks (3). Moreover, oral administration of the zonulin inhibitor (AT-1001) to these rats blocked autoantibody formation, zonulin-mediated IIP, and finally reduced the incidence of diabetes (3). AT-1001 competitively blocks the apical zonulin receptor and prevents the opening of the TJ (2).

In this study, the hypothesis that patients with HT have IIP was investigated. To this end, blood zonulin levels were examined in patients with HT and patients with congenital hypothyroidism (CH) were used as a control group. To the best of our knowledge this is the first study which examines zonulin levels in patients with HT.

## Methods

This was a case-control study in a group of 30 consecutive children and adolescents with HT, and 30 age-, gender- and BMI-matched patients with CH. The diagnosis of HT was based on positive anti-thyroid peroxidase antibody (TPO), anti-TG (antibodies against thyroglobulin) levels and the presence of a typical thyroiditis ultrasound pattern. Obese patients and patients with acute or other chronic diseases were excluded from the study. Informed consent was obtained in accordance with a protocol approved by the Institutional Review Board of Sadi Konuk Training and Research Hospital (2017-207).

### Anthropometric Measurements

Heights were measured in the standing position with bare feet, using Harpenden equipment (Holtain, United Kingdom), and an electronic scale sensitive to 0.1 kilogram (Seca, Germany) was used for weight measurements. Body mass index (BMI) was calculated according to the standard formula. Height, weight and BMI z-scores standard deviation score (SDS) were calculated according to national standards (5). Obesity was defined as a BMI-SDS ≥2 SDS.

### Laboratory Evaluation

Venous blood samples were collected for zonulin, free thyroxine (fT4), thyroid stimulating hormone (TSH), anti-TPO, and anti-TG levels. Samples were immediately processed and sera were stored at -80 °C for subsequent batch analysis. Zonulin levels were measured by human zonulin enzyme-linked immunosorbent assay kit (ELISA, Elabscience, Houston, Texas, USA). Levels of fT4, TSH, anti-TPO and anti-TG were measured by electrochemiluminescence immunoassay (ECLIA, Cobas e602, Roche Diagnostics, Basel, Switzerland).

Thyroid ultrasound results were retrieved from the patient’s files.

### Statistical Analysis

The Shapiro-Wilk test was used to determine whether variables were normally distributed. Data were presented as mean±SDS. Comparisons were made by using independent samples t-test or χ^2^ test. Pearson analysis was used to determine correlations between zonulin levels and other clinical parameters. Separate linear regression models were created to examine the associations of blood zonulin levels in patients with HT and CH. All statistical analyses were conducted with Statistical Package for the Social Sciences version 15.0 (IBM Inc., Chicago, IL., USA). Statistical significance was defined as p≤0.05.

## Results

### Patients’ Characteristics

**Figure 1:**
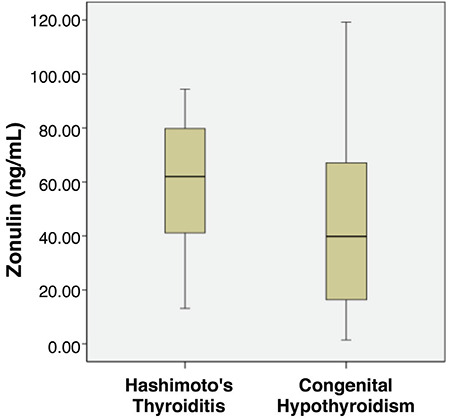
Serum zonulin levels (ng/mL) in patients with Hashimoto thyroiditis and congenital hypothyroidism

Serum zonulin levels were significantly higher in patients with HT than patients with CH (Table 1, Figure 1). Age, gender, weight SDS, height SDS and BMI-SDS were not different between the groups (Table 1). Serum fT4 levels and daily levothyroxine dose were higher in patients with CH, but TSH levels were not different between the groups (Table 1). Anti-TPO and anti-TG levels were higher in patients with HT (Table 1). Thyroid ultrasonography in patients with HT revealed heterogeneous parenchyma in 17 patients and combined heterogeneous and pseudo-nodular parenchyma in 13 (Table 1). In patients with CH, ultrasonography showed agenesis of the thyroid gland in 15 patients, heterogeneous parenchyma in three and normal thyroid gland in 12 patients (Table 1).

### Correlation Analyses

In patients with HT, zonulin levels were positively correlated with weight (r=0.406, p=0.03), weight SDS (r=0.377, p=0.04), BMI (r=0.486, p=0.006), BMI-SDS (r=0.419, p=0.02) and levothyroxine dose (r=0.463, p=0.02). There was no significant correlation between zonulin levels and anti-TPO or anti-TG levels (r=-0.174, p=0.4 and r=0.295, p=0.1, respectively).

In patients with CH, there were strong positive correlations between zonulin levels and age (r=0.475, p=0.008), weight (r=0.707, p<0.001), weight SDS (r=0.532, p=0.002), BMI (r=0.872, p<0.001), BMI-SDS (r=0.681, p<0.001) and levothyroxine dose (r=0.485, p=0.007).

### Regression Analyses

In patients with HT, serum zonulin was only associated with levothyroxine dose after adjusting for age, weight, TSH and fT4 levels, but with a borderline p-value (R^2^=0.36, p=0.05).

When the patients with CH were put into the same regression model, there was no significant association between zonulin level and levothyroxine dose (p=0.4). However, serum zonulin was strongly associated only with weight in these patients (R^2^=0.62, p<0.001).

## Discussion

Intestinal mucosa is the largest contact site for the host’s immune system and exterior antigens, such as food antigens, bacteria, pathogens, and toxins (6). Increase in permeability can cause an exposure of sub-mucosal immune cells to these various antigens and may lead to the development of autoimmune disorders (6,7). Indeed, previous studies have shown that the increased permeability is a key pathogenic component rather than an epiphenomenon of autoimmune disorders (3,4). It was hypothesized that intestinal permeability is also increased in HT (8), but to the best of our knowledge, to date, no study has addressed this issue in these patients. Only in a letter to the editor, Sasso et al (9) described ultrastructural morphological changes of distal duodenum enterocytes in four patients with HT. They also reported IIP, evaluated by a lactulose/mannitol test, in these four patients (9). In our study, it has been demonstrated that children and adolescents with HT had increased zonulin levels compared to their age, gender and BMI matched peers with CH.

Intestinal TJ create gradients for the optimal absorption of nutrients and control tolerance/immunity balance to non-self antigens (10). Zonulin, a physiological modulator of TJ, is involved in trafficking of macromolecules and, therefore, in balance between tolerance and immune response (2,11). Sturgeon et al (12) identified zonulin as a master regulator of intercellular TJ. They showed that, there was IIP present in zonulin transgenic mice compared to wild-type mice, which was associated with upregulation of zonulin gene expression. Moreover, treatment with AT1001 (Larazotide acetate) reduced intestinal permeability both *in vivo* and *ex vivo* and reverted morbidity and mortality in these zonulin transgenic mice. Their data demonstrated that zonulin-dependent small intestinal barrier dysfunction is an early step leading to the break of tolerance with subsequent development of colitis (12). Similarly, Arrieta et al (13) reported that IL10 gene-deficient mice treated with AT-1001, showed a marked decrease in small intestinal permeability and a clear reduction of colitis severity. In addition, several clinical trials reported a potential beneficial effect of AT-1001 in patients with celiac disease (14). In a study of 339 type 1 diabetic patients and their first degree relatives, Sapone et al (4) showed that patients with type 1 diabetes and their relatives have elevated serum zonulin levels that correlate with IIP associated with altered genetic expression of intestinal TJ proteins. Zonulin upregulation was shown to precede the onset of diabetes, providing a possible link between IIP, environmental exposure to non-self antigens, and the development of autoimmunity in genetically susceptible individuals for type 1 diabetes (4). Our finding of increased zonulin levels in patients with HT may trigger future studies that may lead to potential new therapies for HT.

Increased zonulin levels have been reported with aging and obesity (15). It has been suggested that IIP may play a critical role in the development of age-related and obesity-related inflammation and comorbidities. Similarly, we showed that, serum zonulin levels were strongly correlated with age, weight and BMI, in our study group.

Serum zonulin levels were also positively correlated with levothyroxine dose both in patients with CH and HT. Moreover, in patients with HT, zonulin level was only associated with levothyroxine dose after adjusting for age, weight, TSH and fT4 levels. By contrast, there was no such association in CH patients. This finding might imply a relationship between serum zonulin levels and disease severity in HT, because in patients with CH, levothyroxine dose is mainly based on the amount of residual thyroid tissue/function, age, body weight, and thyroid hormone levels (16). However, in patients in HT, there is another associate of the levothyroxine dose; degree of the thyroid damage by autoimmune mechanisms (17). We did not find a significant correlation between anti-TPO or anti-TG antibodies and serum zonulin levels. However, the contribution of anti-TPO antibodies to thyroid damage compared to T-cell and cytokine-mediated apoptosis in HT is minor and anti-TG antibodies do not cause thyroid cell destruction (18,19). Thyroid damage in HT is mostly due to an apoptotic processes combined with CD8+ cell mediated cytotoxicity, changes in cell junctions, and complement activation (18,19).

### Study Limitations

This was a pilot study with small numbers of children. In addition, we did not have a healthy control group to compare zonulin levels in children and adolescents who had normal thyroid function and therefore would not be on levothyroxine therapy.

## Conclusion

Higher zonulin levels in children and adolescents with HT suggested IIP in these patients. Further research is needed to expand these results in larger cohorts to gain more insight into the pathogenesis of HT and to lead to potential new treatments.

## Figures and Tables

**Table 1:**
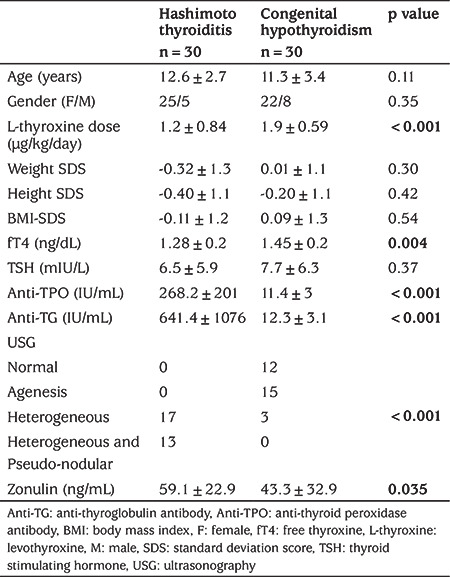
Comparison of the clinical and laboratory parameters of the patients with Hashimoto thyroiditis and with congenital hypothyroidism (all values are means±standard deviations, except if otherwise stated)
